# Single mitochondrial gene barcodes reliably identify sister-species in diverse clades of birds

**DOI:** 10.1186/1471-2148-8-81

**Published:** 2008-03-09

**Authors:** Erika S Tavares, Allan J Baker

**Affiliations:** 1 Department of Natural History, Royal Ontario Museum, 100 Queen's Park, Toronto, Canada; 2 Department of Ecology and Evolutionary Biology, University of Toronto, Toronto, Canada

## Abstract

**Background:**

DNA barcoding of life using a standardized COI sequence was proposed as a species identification system, and as a method for detecting putative new species. Previous tests in birds showed that individuals can be correctly assigned to species in ~94% of the cases and suggested a threshold of 10× mean intraspecific difference to detect potential new species. However, these tests were criticized because they were based on a single maternally inherited gene rather than multiple nuclear genes, did not compare phylogenetically identified sister species, and thus likely overestimated the efficacy of DNA barcodes in identifying species.

**Results:**

To test the efficacy of DNA barcodes we compared ~650 bp of *COI *in 60 sister-species pairs identified in multigene phylogenies from 10 orders of birds. In all pairs, individuals of each species were monophyletic in a neighbor-joining (NJ) tree, and each species possessed fixed mutational differences distinguishing them from their sister species. Consequently, individuals were correctly assigned to species using a statistical coalescent framework. A coalescent test of taxonomic distinctiveness based on chance occurrence of reciprocal monophyly in two lineages was verified in known sister species, and used to identify recently separated lineages that represent putative species. This approach avoids the use of a universal distance cutoff which is invalidated by variation in times to common ancestry of sister species and in rates of evolution.

**Conclusion:**

Closely related sister species of birds can be identified reliably by barcodes of fixed diagnostic substitutions in *COI *sequences, verifying coalescent-based statistical tests of reciprocal monophyly for taxonomic distinctiveness. Contrary to recent criticisms, a single DNA barcode is a rapid way to discover monophyletic lineages within a metapopulation that might represent undiscovered cryptic species, as envisaged in the unified species concept. This identifies a smaller set of lineages that can also be tested independently for species status with multiple nuclear gene approaches and other phenotypic characters.

## Background

Large scale sequencing of a predefined region of approximately 650 (base pairs) bp of the mitochondrial gene *COI*, known as DNA barcoding, has two main goals: 1) to develop a species identification system that also allows unknown individuals to be assigned to species; 2) and to enhance the discovery of new species [1-3]. Although DNA barcoding has proved effective in achieving both goals in several large groups of animals [4-11], the efficacy of the tests have been questioned [12-16].

A major test performed on 643 previously recognized species of birds of North America demonstrated the effectiveness of DNA barcoding because 94% possessed unique monophyletic *COI *clusters [10,11]. The remaining 6% of the species did not have unique DNA barcodes, indicating that they either were (a) wrongly identified in the past as separate species, (b) closely related species that hybridize regularly, or (c) species losing identity by secondary contact [11]. These groups may be in the indeterminate zone between differentiated populations and distinct species [10,11]. Critics of DNA barcoding claim that in spite of the impressive number of bird species sampled [11], the precision of the method was compromised due to insufficient intraspecific sampling, and because comparisons among species were not exclusively from sister-species pairs [12,15,17], where taxonomic uncertainty, interspecific hybridization, and incomplete lineage sorting could decrease the effectiveness of the test [12]. The suggested threshold of 10 times the mean intraspecific variation (10 × rule) to screen for splits referred to as 'putative' species [11] has also been criticized. Moritz and Cicero [12] reported significantly lower average mitochondrial DNA distances between sister species of birds than levels reported in the barcoding tests of birds [10,11], although the distances from these sister-species comparisons came from a variety of methods and genes [7]. Meyer and Paulay [13] tested different threshold methods in *COI *barcodes of cowries and found extensive overlap of overall intraspecific distances with interspecific distances, resulting in minimum error rates of ~17% to screen for putative new species. Additionally, a simulation study using the neutral coalescent and the Bateson-Dobzhansky-Muller (BDM) model of speciation suggested that mtDNA barcodes will have error rates lower than 10% in assigning individuals to species only when populations have been isolated for more than 4 million generations [15]. A universal-distance cutoff is therefore not an objective criterion to delineate species limits [18].

Additionally, Hickerson et al. [15] argued that reciprocal monophyly of mtDNA sequences and the 10 × threshold will likely underestimate species diversity [15]. Tree-based approaches with genetic distances that use reciprocal monophyly for species delimitation can be problematic because aggregations of haplotypes in phylogenetic trees, even when highly supported, do not necessarily imply that they belong to a distinctive taxonomic unit [19]. To address these issues, Rosenberg [19] proposed a statistical test to test if monophyletic groups in a phylogenetic tree are more likely to represent distinctive taxonomical entities, or are just random branches of lineages within a species. This approach also suggests minimal sample sizes required for inferences to be made about taxonomic distinctiveness from observations of monophyly [19].

Some of the advantages of using a single mtDNA barcode to identify species are that it has a higher rate of evolution (and thus more mutations), and because matrilineal lineages sort into reciprocally monophyletic clades much faster than nuclear genes [20]. This reduces the incidence of incompletely sorted lineages relative to that expected with nuclear genes. However, recent simulations with multiple nuclear genes indicate that very recently derived species can be identified well before the time to reciprocal monophyly [21]. Additionally, species were correctly delimited in <50% of replicates simulating mtDNA sequences, suggesting that the single gene barcode approach was insufficient to delimit recently diverged species.

In response to the above criticisms we initiated a more comprehensive study of 60 sister-species pairs of birds defined rigorously with multigene phylogenies to determine whether mtDNA barcodes can reliably distinguish closely related sister species. Instead of the much criticized 10× rule, which may not apply in recently diverged sister-species pairs, we use coalescent-based statistical tests for species distinctiveness under reciprocal monophyly [19]. Additionally, we show that even recently diverged sister-species pairs have fixed nucleotide substitutions that serve as diagnostic mtDNA barcodes envisioned in the original analogy. Such diagnostic barcodes are useful not only in quickly identifying known species of birds but also in flagging other recently derived evolutionary lineages that could be analyzed with multilocus methods [21-23] to determine if they represent emergent species.

## Results

### DNA barcodes distinguish sister-species of birds

Monophyletic clusters of individuals corresponding to species were recovered in a Neighbor-joining (NJ) tree under the Kimura 2-parameter (K2P) model in all the sister-species pairs compared (Table 1, see Additional files 1, 2). Multiple diagnostic characters in the branches of the trees leading to species clusters were detected in all the pairs (see Additional file 1, Figure 1). Bootstrap support at the nodes grouping individuals of the same species varied from 55 to 100%, except for Eastern Meadowlark (*Sturnella magna*), with the majority of the values (93.1%) above 85% (see Additional file 1). Species with clusters of individuals supported with bootstrap levels below 85% were: Ruby-throated Hummingbird (*Archilochus colubris*), Black-chinned Hummingbird (*Archilochus alexandri*), Gunnison Sage-Grouse (*Centrocercus minimus*), Dusky Grouse (*Dendragapus obscurus*), Nuttall's Woodpecker (*Picoides nuttallii*), Jackass Penguin (*Spheniscus demersus*), and Magellanic Penguin (*Spheniscus magellanicus*). These species were distinguished by <10 fixed nucleotide substitutional differences or had multiple intraspecific clusters. Probabilities of chance occurrence of reciprocal monophyly arising from random-branching within a single taxon were smaller than the level of significance (α) of 5% (Table 1). Ideally, larger sample sizes are required to increase the power of the test and to confirm reciprocal monophyly over a broad geographic range.

**Figure 1:**
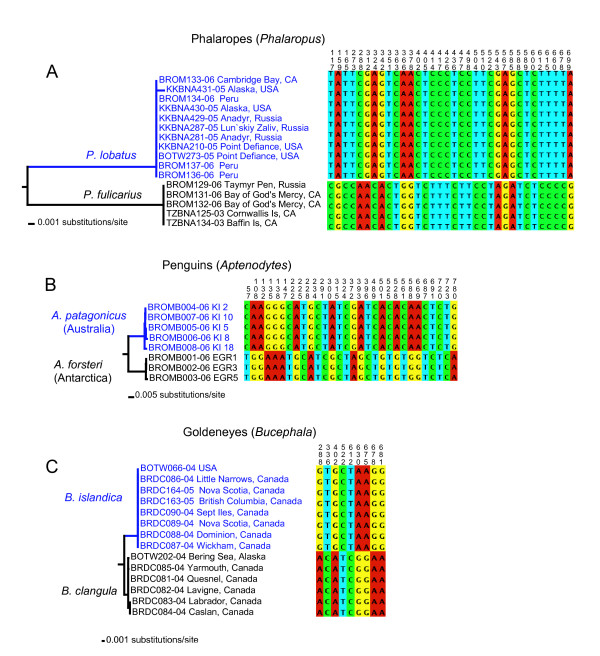
**Examples of DNA barcodes distinguishing sister species**. Neighbor-joining tree constructed with K2P genetic distances. Fixed substitutions are represented by coloured boxes, with corresponding character positions relative to the beginning of *COI*. a) Phalaropes (*Phalaropus*); b) Penguins (*Aptenodytes*); c) Goldeneyes (*Bucephala*).

**Table 1:** Comparisons of sister-species pairs from some major clades of birds. Sister-species pairs, probability of chance reciprocal phylogeny (p), and reference for phylogenetic relationship (r).

Sister-species pairs		p	r	Sister-species pairs		p	r
*Acridotheres tristis*	*A. ginginianus*	4.7 × 10^-6^	[52]	*Melospiza lincolnii*	*M. georgiana*	9.7 × 10^-5^	[53]
*Actitis hypoleucos*	*A. macularius*	2.3 × 10^-4^	[47]	*Mitu tuberosum*	*M. salvini*	5.0 × 10^-2^	[54]
*Aethia cristatella*	*A. psittacula*	4.1 × 10^-3^	[48]	*Molothrus bonariensis*	*M. aeneus*	6.1 × 10^-4^	[55]
*Aethia pygmaea*	*A. cristatella*	4.1 × 10^-3^	[48]	*Morus capensis*	*M. serrator*	5.1 × 10^-3^	[56]
*Aethia pygmaea*	*A. psittacula*	4.1 × 10^-3^	[48]	*Numenius phaeopus*	*N. tahitiensis*	8.8 × 10^-4^	a
*Aptenodytes forsteri*	*A. patagonicus*	5.1 × 10^-3^	[49]	*Passerina ciris*	*P. versicolor*	5.0 × 10^-2^	[57]
*Apteryx haastii*	*A. owenii*	2.0 × 10^-2^	[50]	*Phalaropus fulicarius*	*P. lobatus*	3.1 × 10^-5^	a
*Apteryx mantelli*	*A. rowi*	4.2 × 10^-4^	[50]	*Pheucticus melanocephalus*	*P. ludovicianus*	1.9 × 10^-3^	[58]
*Archilochus colubris*	*A. alexandri*	5.1 × 10^-3^	[59, 60]	*Picoides nuttallii*	*P. scalaris*	2.7 × 10^-2^	[61]
*Brachyramphus brevirostris*	*B. marmoratus*	7.6 × 10^-5^	[48]	*Puffinus bulleri*	*P. pacificus*	2.0 × 10^-4^	[62]
*Bubo virginianus*	*B*. *scandiacus*	1.0 × 10^-5^	[63]	*Pygoscelis antarcticus*	*P. papua*	1.4 × 10^-5^	[49]
*Bucephala clangula*	*B. islandica*	5.1 × 10^-5^	[64]	*Recurvirostra americana*	*R. andina*	6.9 × 10^-3^	[65]
*Calamospiza melanocorys*	*Chondestes grammacus*	1.6 × 10^-2^	[53]	*Rhea pennata*	*R. americana*	9.5 × 10^-3^	[66]
*Calcarius pictus*	*C. ornatus*	6.9 × 10^-3^	[67]	*Rissa brevirostris*	*R. tridactyla*	2.8 × 20^-3^	[48]
*Tringa semipalmata*	*T. flavipes*	2.2 × 10^-5^	[47]	*Spheniscus demersus*	*S. magellanicus*	9.5 × 10^-3^	[49]
*Centrocercus minimus*	*C. urophasianus*	1.6 × 10^-2^	[68]	*Sialia currucoides*	*S. mexicana*	2.3 × 10^-4^	[69]
*Chlidonias leucopterus*	*C. niger*	1.6 × 10^-2^	[46]	*Sialia sialis*	*S. mexicana*	4.5 × 10^-5^	[69]
*Cyanocitta cristata*	*C. stelleri*	5.1 × 10^-5^	[70]	*Spizella breweri*	*S. passerina*	1.2 × 10^-6^	[53]
*Dendragapus fuliginosus*	*D. obscurus*	5.0 × 10^-2^	[71]	*Spizella pallida*	*S. breweri*	7.7 × 10^-5^	[53]
*Eudyptes pachyrhynchus*	*E. robustus*	2.7 × 10^-2^	[49]	*Stercorarius parasiticus*	*S. longicaudus*	6.4 × 10^-6^	[72]
*Euphagus carolinus*	*E. cyanocephalus*	1.9 × 10^-3^	[73]	*Hydroprogne caspia*	*Gelochelidonnilotica*	6.4 × 10^-6^	[46]
*Fratercula arctica*	*F. corniculata*	6.1 × 10^-4^	[48]	*Thalasseus sandvicensis*	*T. elegans*	5.1 × 10^-5^	[46]
*Cerorhinca monocerata*	*Fratercula cirrhata*	3.0 × 10^-3^	[48]	*Sternula antillarum*	*S. superciliaris*	9.5 × 10^-3^	[46]
*Tringa brevipes*	*T. incana*	2.0 × 10^-2^	[47]	*Sturnella neglecta*	*S. magna*	2.3 × 10^-4^	[55]
*Himantopus himantopus*	*Hi. leucocephalus*	3.0 × 10^-3^	[65]	*Sturnia malabarica*	*Temenuchus pagodarum*	1.0 × 10^-2^	[52]
*Himantopus melanurus*	*Hi. mexicanus*	9.5 × 10^-3^	[65]	*Synthliboramphus antiquus*	*S. wumizusume*	2.7 × 10^-2^	[48]
*Icterus galbula*	*I. bullockii*	4.3 × 10^-4^	[74]	*Toxostoma rufum*	*T. curvirostre*	1.0 × 10^-2^	[52]
*Jacana spinosa*	*J. jacana*	5.0 × 10^-2^	[75]	*Tringa glareola*	*T. totanus*	2.2 × 10^-5^	[47]
*Lagopus muta*	*L. lagopus*	1.2 × 10^-6^	[76]	*Tringa melanoleuca*	*T. nebularia*	2.3 × 10^-4^	[47]
*Limnodromus griseus*	*L. scolopaceus*	1.9 × 10^-3^	[77]	*Uria aalge*	*U. lomvia*	2.3 × 10^-4^	[48]

a- genus comprising few species.

### Individuals were correctly assigned to their corresponding species

**Figure 2:**
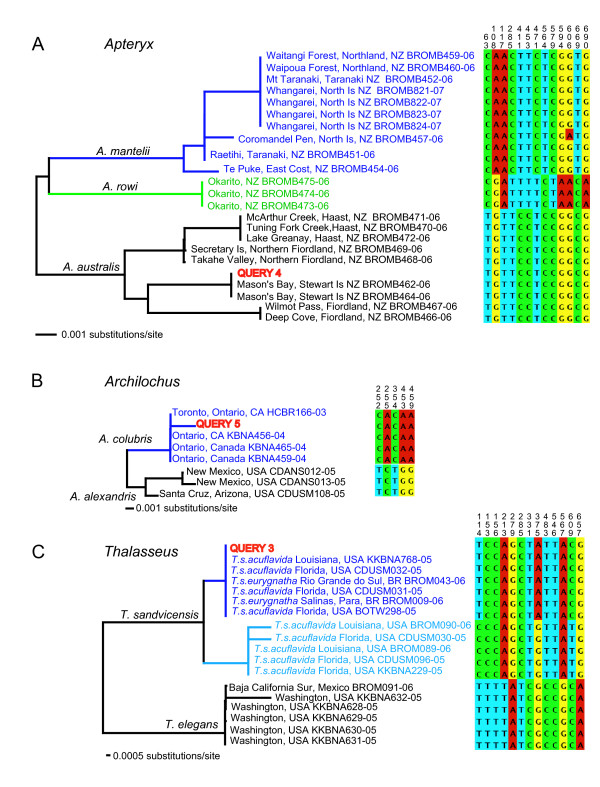
**Assignment of unknowns**. Neighbor-joining tree constructed with K2P genetic distances. Fixed substitutions are represented by coloured boxes, with corresponding character positions relative to the beginning of *COI*. Query specimens used in the test of assignment are indicated in red, with additional information in Table 2. a) Okarito Brown Kiwi (*Apteryx rowi*); b) Ruby-throated Hummingbird (*Archilochus colubris*); c) Sandwich Tern (*Thalasseus sandvicensis*).

Individuals from the six species-pairs with adequate samples sizes were picked randomly to query whether they could be assigned correctly to their species using clustering in a NJ tree, fixed mutations, and a statistical test of assignment based on coalescent theory [24] (Table 2, Figure 2). In all the cases the query individual was correctly assigned to species with posterior probability of 1.0 and correspondingly tiny risk of misassignment (Table 2, Figure 2). When species barcodes were comprised of more than one intraspecific cluster, as in Southern Brown Kiwi (*Apteryx australis*, Figure 2A), Gull-billed Tern (*Gelochelidon nilotica*) and Gentoo Penguin (*Pygoscelis papua*), the query individual was assigned correctly to the each intraspecific cluster (Table 2).

**Table 2:** Assignment of individuals to species. Query individual to be assigned, specimen details, diagnostic sites, posterior probability of assignment (Post. prob.), and risk of mis-assignment.

Query Species	Specimen ID.	Collecting locale	Diagnostic sites (#)	Post. prob.	Risk
1. Common Goldeneye *Bucephala clangula*	1510–10045	Labrador, Canada	9	1	1.815 × 10^-46^
2. Lincoln's Sparrow *Melospiza lincolnii*	KKBNA111-4 CWSL94-65761-04	Saskatchewan, Canada	9	1	6.661 × 10^-34^
3. Sandwich Tern *Thalasseus sandvicensis*	KKBNA472-05 UWBM 73832	Louisiana, USA	11	1	4.315 × 10^-31^
4. Okarito Brown Kiwi *Apteryx rowi*	BROMB463-06 RA 0886	Stewart Is., New Zealand	13	1	1.568 × 10^-37^
5. Ruby-throated Hummingbird *Archilochus colubris*	TZBNA028-03 1B-269	Ontario, Canada	7	1	1.946 × 10^-16^
6. Gentoo Penguin *Pygoscelis papua*	GPB1	Falkland Islands	35	1	2.831 × 10^-99^

### Species level delimitation with the "10 × rule"

**Figure 3:**
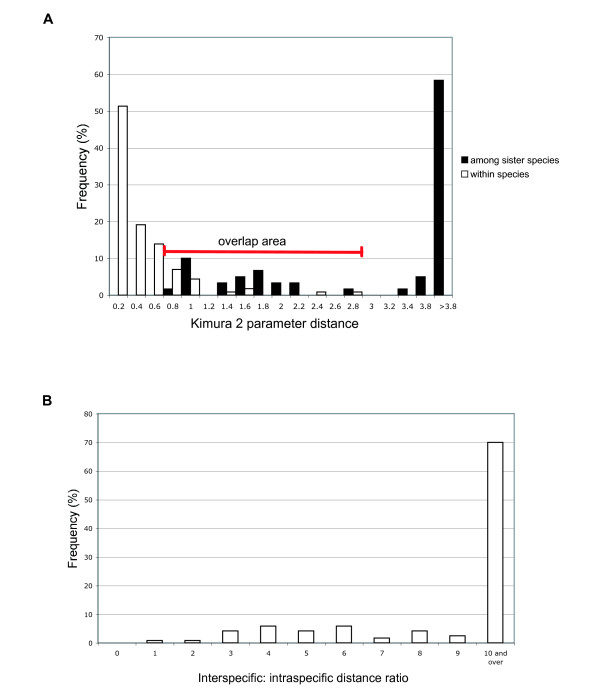
**Levels of intraspecific and interspecific distances of sister-species of birds**. a) Frequency distribution of K2P intraspecific and interspecific genetic distances between sister-species. b) Frequency distribution of the ratios of K2P interspecific: intraspecific distances in sister species of birds.

Mean among sister-species distances of mtDNA barcodes varied from 0.78% to 11.77%, with 20 out of 60 (28.6%) distances smaller than the 2.7% threshold used to flag potential new species of birds. Among-species distances overlapped maximum within-species distances in 39 of 60 (65%) sister-species pairs. Excluding cases that are likely to represent overlooked species based on other attributes, the overlap was observed in 21 of 60 sister-species pairs (35%, Figure 3A). However, *COI *sequences in several species were structured in NJ trees into clades that represent geographically structured populations, recognized subspecies or possibly cryptic species (Table 3). The ratios of among-species to within-species distances were above 1 except for western and eastern populations of Eastern Meadowlark (*Sturnella magna*) which are thought to be two species [11,25] (Figure 3B).

**Table 3:** Possible taxonomically distinctive entities. Intraspecific clusters of individuals that might be unrecognized species, probability of chance reciprocal monophyly (p, α ≤ 0.01), specimen details, fixed diagnostic mutations, and mean distances between the clusters of the same species.

Species	p	Collecting locale or subspecies (sampling)	Fixed mutations	Mean D (%) among clusters
*Brachyramphus brevirostris*	3.0 × 10^-3^	a. Aleutians, Russia (3)	a vs b = 7	a vs b = 1.23
		b. East Alaska (6)		
*Pygoscelis papua*	9.7 × 10^-5^	a. Macquarie Island (6)	a vs b = 15	a vs b = 2.43
		b. Falkland Island (7)		
*Gelochelidon nilotica*	1.8 × 10^-3^	a. Small form of the beak (3)	a vs b = 11	a vs b = 1.74
	9.5 × 10^-3^	b. Large form of the beak (3)	a vs c = 10	a vs c = 1.84
		c. South America, Russia (4)	b vs c = 5	b vs c = 1.74
*Sturnella magna*	9.5 × 10^-3^	a. Texas (4)	a vs b = 22	a vs b = 4.03
		b. Texas, Ontario, Miami (3)		
*Tringa totanus*	9.5 × 10^-3^	a. Iceland (4)	a vs b = 6	a vs b = 0.95
		b. Vietnam, Australia (3)		
*Eudyptula minor*	8.3 × 10^-17^	a. New Zealand (NZ)(21)	a vs b = 28	a vs b = 3.82
		b. Australia (21)		

**Figure 4:**
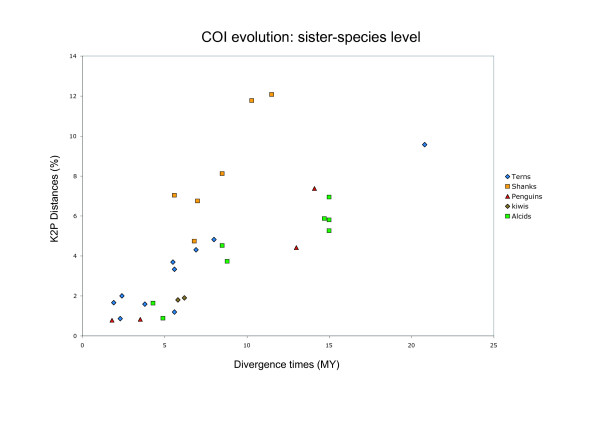
**Variable rates of *COI *evolution in different lineages of birds**. lot of the K2P genetic distances among sister-species versus divergence times obtained from chronograms of different clades of birds.

**Figure 5:**
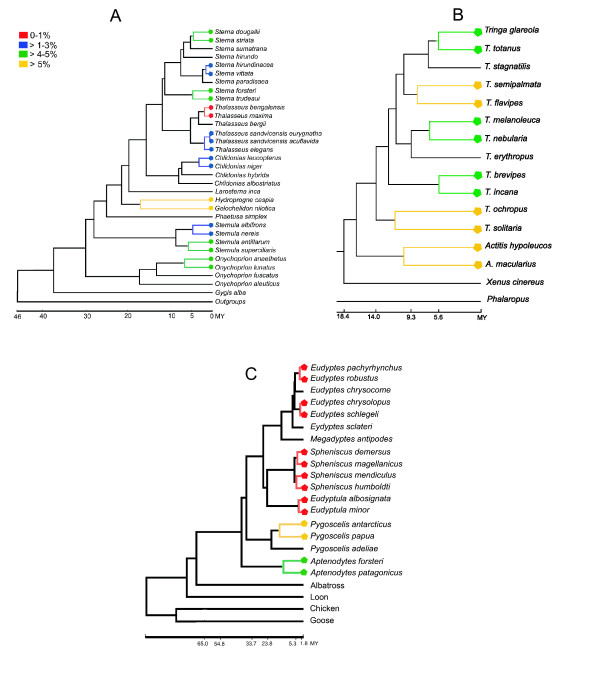
**K2P distances of DNA barcodes mapped on chronograms of different lineages of birds**. hronograms of diverse clades of birds. Legend correspond to K2P divergence levels in DNA barcodes : a) terns; b) shanks, and c) penguins.

Plots of corrected *COI *distances against divergence times revealed that mutations are accumulating roughly linearly in all the groups we evaluated (Figure 4). However, the rates of evolution are variable. For example, shanks accumulate more mutations in *COI *than do terns and penguins per unit time (Figures 4, and 5A–C). Variation in rates of evolution of *COI *in different clades of birds mitigates against a universal distance criterion for species recognition, in accordance with previous evidence from a mitogenomic timescale for birds [26].

### Intraspecific variation suggesting potential distinctive taxonomical entities

**Figure 6:**
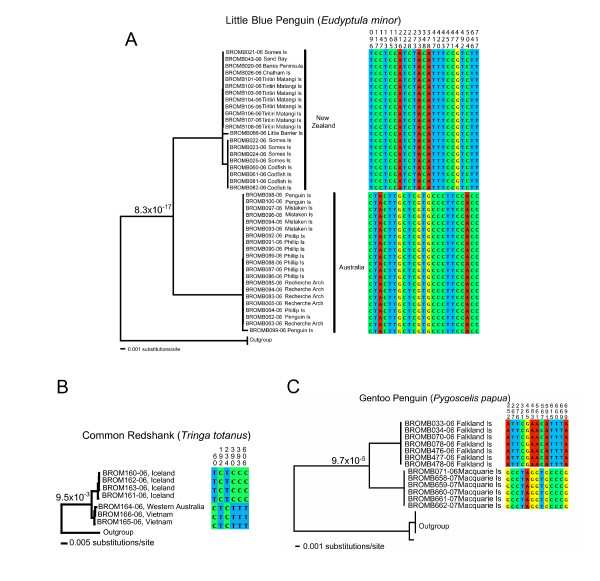
**Reciprocally monophyletic groups possibly indicating unrecognized species**. eighbor-joining tree constructed with K2P genetic distances. Fixed substitutions are represented by coloured boxes, with corresponding character positions relative to the beginning of *COI*. a) Little Penguin (*Eudyptula minor*); b) Common Redshank (*Tringa totanus*) and, c) Gentoo Penguin (*Pygoscelis papua*).

Six species had distinctive intraspecific clusters with probabilities of chance reciprocal monophyly below a conservative level of α = 1%: Kittlitz's Murrelet (*Brachyramphus brevirostris*), Gentoo Penguin (*Pygoscelis papua*), Gull-billed Tern (*Gelochelidon nilotica*), Eastern Meadowlark (*Sturnella magna*), Common Redshank (*Tringa totanus*), and Little Penguin (*Eudyptula minor*, Table 3, Figure 6). These groups represent recognized subspecies, populations occupying different geographical areas or distinct morphotypes. DNA barcode sequences of *Gelochelidon nilotica *comprised three intraspecific clusters in NJ trees (Figure 6C, Table 3). Two of the groups had discontinuous beak size distributions (pers. obs.) that were thought to represent Australian and Asian subspecies *S. n. macrotarsa *and *S. n. affinis*, respectively [27]. The other group comprised reciprocally monophyletic lineages representing the subspecies *S. n*. *groenvoldi *(South America) and *S. n. vanrossemini *(Russia), but they were poorly sampled (2 samples each) [28].

Using the test for chance reciprocal monophyly, the Little Penguins of Australia and New Zealand, respectively, currently lumped into *Eudyptula minor*, are probably two species (Table 3). This conclusion is supported by a high number of fixed differences in the DNA barcodes and in multigene phylogenies [29] (Table 3, Figure 6A). Other species are comprised of monophyletic groups that could be taxonomically distinctive, although the probabilities of chance reciprocal monophyly are between 1–5%. For example, specimens of Australasian Pipit (*Anthus novaeseelandiae*) from New Zealand and Australia differ by 4.1% in their barcodes, and Little Terns (*Sterna albifrons*) from England and Australia differ by about 1%. However, increased sampling of these species is required to properly test whether they represent separate taxonomic entities.

## Discussion

### Effectiveness of single gene *COI *barcodes

Our study of 60 pairs of sister species from a broad range of bird clades showed that closely related pairs could not be distinguished using the 10× rule of among to within species divergence, as predicted by critics of this criterion [12,15]. Similarly, the suggested threshold genetic distance of 2.7% to flag potential species failed to detect recently evolved sister species, and was further compromised by substantial variation in the rate of *COI *evolution in different clades and short species divergence times. However, all sister-species pairs were shown to possess unique DNA barcodes by which they could be identified. In particular, the *COI *sequences of even very closely related sister species were found to have diagnostic combinations of 5–64 fixed substitutional differences that better fit the analogy of a short DNA barcode. Individuals were correctly assigned to each sister species for which we had moderate sample sizes (N ≥ 4) using different lines of evidence: NJ clustering, diagnostic fixed substitutions, and a decision-theoretic framework based on coalescent theory implemented in Assigner [24]. The concern about assigning taxonomically unknown specimens to an existing or new taxon is unlikely to be a serious problem in birds, given the uniqueness of species barcodes and the mature taxonomy of the clade.

Phylogroups of *COI *sequences representing within-species variation can potentially be confounded with recently diverged sister species, so to objectively discriminate between these two possibilities we applied a statistical test of the null hypothesis that reciprocal monophyly has arisen by random branching of lineages within a single species. The null hypothesis could be rejected in all closely related sister species (*P *< 0.05), verifying the power of the test. In addition, putative new species were strongly supported by the distinctive signatures of >12 fixed substitutional differences and low probabilities of chance reciprocal monophyly within a single species. For example, the barcodes of Little Penguins from Australia and New Zealand, and of Gentoo Penguins from Macquarie Island and the Falklands, provide strong inferences of separate lineages that may warrant species status for these groups. The existence of separately evolving metapopulation lineages is the species delimitation criterion for a recently proposed unified species concept [30], though contingent properties such as phenetic, behavioural and reproductive differences need to be assessed in future to provide additional lines of evidence for or against species status. This is not a weakness of a single mtDNA gene barcoding system as has been claimed [21], but rather is a rapid way to discover monophyletic lineages within a metapopulation that might represent undiscovered cryptic species. The barcoding approach used here can be applied to other organismal groups where individuals of the same species cluster in monophyletic clades despite overlaps in within- and among-species variation [14]. However, will not be applicable in groups with no mitochondrial divergence observed between species pairs (ex. [31]).

### Single gene versus multilocus approaches for species delimitation

One of the most cogent criticisms of single locus mtDNA barcodes is that a pattern of reciprocal monophyly in maternally inherited genes can also arise when female dispersal is very restricted, often contrasting with widespread apparent panmixia of autosomal and paternally inherited genes [32]. However, if sister species have diverged very recently then sufficient time may not have passed for enough mutations in a nuclear gene to have accumulated to reliably track lineage splitting and resolve problems with incomplete sorting of ancestral polymorphism. This in turn can lead to erroneous inference of extensive gene flow in autosomal genes if it is based on single gene trees. In such situations use of multiple nuclear genes is increasingly being touted to help delimit species boundaries [21-23]. Recent simulations in a coalescent-based approach showed that species limits were delimited with high probability depending on the number of loci examined and the timing of species divergence [21]. Ten loci were able to reliably detect species with effective population sizes of 100,000 that diverged in a timeframe (31,000 generations ago) when incomplete lineage sorting would be expected to occur. Obviously, this multilocus approach is currently infeasible for the purpose of barcoding life on the planet, but it will be invaluable for inferring species limits in very recently separated species pairs where mtDNA barcodes alone might not be definitive. The 60 previously identified sister-species pairs of birds we studied had unique mtDNA barcodes that identified them, and each species was characterized by fixed mutational differences that are unlikely to be reduced substantially in number by increased sampling of polymorphic sites. However, species in which well differentiated reciprocally monophyletic clades of *COI *haplotypes were detected would seem to be fertile ground for further investigation with independent multiple nuclear gene trees in a coalescent framework. For example, the split between Australian and New Zealand populations of Little Penguins was dated at approximately 1.3 Mya using the neutral coalescent method in IM [33], and a phylogenetic rate of *COI *evolution of 0.01354 substitutions/site/Myr [26]. Given a generation time of 6.5 years (based age of first breeding of 2.5 years and annual survival of breeding adults 80% [34] this equates roughly to 200,000 generations, where incomplete lineage sorting of autosomal genes should be reduced unless effective population size is very large [35]. The faster sorting of *COI *sequences might be an advantage in identifying possible recent speciation events, and they can be combined with nuclear gene sequences in IM to estimate whether the divergence is due to isolation or if gene flow has been ongoing. Thus we view DNA barcodes as useful complements in multigene data sets that might include more than one mtDNA gene [36], contrary to recent criticisms of maternally inherited genes in species delimitation.

## Conclusion

We show that in a broad range of birds even closely related sister species delimited with independent evidence could be identified with mtDNA barcodes and diagnostic substitutions using standard *COI *sequences. All pairs were characterized by reciprocally monophyletic lineages, and tests of the null hypothesis of random branching within a single species were rejected. Thus in well studied groups like birds, mtDNA barcodes are extremely effective in identifying sister species. In species that are shown by *COI *barcodes to be comprised of several divergent monophyletic lineages that might flag unrecognized species, it is important to test these splits with multiple independent gene trees in a coalescent framework to guard against the alternative inference of population subdivision via restricted female dispersal. Combination of multiple genes including mtDNA barcodes should counter any biases in species detection and the high variance in associated genetic processes [21].

## Methods

### Taxon sampling

To evaluate the performance of *COI *barcoding in detecting species boundaries of birds we analyzed sister-species pairs defined rigorously by previous phylogenetic studies (Table 1). We excluded species that were known to hybridize to prevent confusion due to introgression, a problem that plagues all methods of species delimitation. In addition, we included species of birds with multiple clusters that might represent unrecognized species. The *COI *sequences generated and used in this work are deposited in the project "Royal Ontario Museum- Birds 1" in the Completed Projects selection of the Barcode of Life Data System (BOLD [37], Genbank Accession numbers EU525241–EU525592). *COI *sequences obtained from previous work are available in the Completed Projects selection of the BOLD, in the "Birds of North America" project [10,11] (Genbank Accession numbers DQ432694–DQ433261, DQ433274–DQ433846, DQ434243–DQ434805).

### DNA extraction and sequencing

DNA was extracted from blood, muscle or liver by phenol, chelex or a membrane purification procedure with glass fiber filtration plates (Acroprep 96 Filter Plate- 1.0 μm Glass, PALL Corporation [38]). PCR amplification of the 5' end of the *COI *gene were performed in a 12.5 μL reaction, with a buffer solution containing 10 mM Tris-HCl, pH 8.3, 50 mM KCl, 2.5 mM MgCl_2_, 0.01% gelatin, and 160 μg/ml bovine serum albumin (BSA) [39], 0.4 mM dNTPs, 0.2 μM of each primer, 1 U *Taq *polymerase (Invitrogen), and 20–25 ng of DNA. Cycle conditions were 36 cycles of 94°C for 40 s, 50°C for 40s, and 72°C for 1 m, with an initial denaturation of 94°C for 5 m and a final extension at 72°C for 7 m. Bird universal primers used were as follows: LTyr – TGTAAAAAGGWCTACAGCCTAACGC, (Oliver Haddrath, pers. comm.) and COI907aH2 – GTRGCNGAYGTRAARTATGCTCG, (Rebecca Elbourne, pers. comm.) Amplified segments were purified by excising bands from agarose gels and centrifuging each through a filter tip. Sequences were obtained on an ABI3100 (Applied Biosystems) according to the manufacturers' suggested protocols using the internal primers COIaRt (forward-AACAAACCACAAAGATATCGG, Oliver Haddrath, pers comm.) and COI748Ht (reverse-TGGGARATAATTCCRAAGCCTGG), or alternatively LTyr (primer used in amplification) and COI745h2 (reverse-ACRTGNGAGATRATTCCRAANCCNG, Rebecca Elbourne, pers. comm.). Sequences were checked for ambiguities in Sequencher 4.1.2 (GeneCodes Corp., Ann Arbor, Michigan) and the multiple alignments was performed in MacClade 4 [40].

### Species delimitation with DNA barcodes

To check for reciprocal monophyly in sister-species with DNA barcodes, a Neighbor-Joining (NJ) tree was constructed in PAUP 4.10b [41] with the Kimura 2 parameter model (K2P). Statistical support was estimated with 1,000 bootstrap replicates in a heuristic search using stepwise addition with 10 random additions of sequences.

Because compound diagnostic characters are a valuable source of information to diagnose species [18] we filtered variable characters for each sister-species pairs in PAUP 4.10b [41], and fixed substitutions were selected in MacClade 4 [40].

The test for chance occurrence of reciprocal monophyly [19] was applied to the sister-species pairs with α = 5%. We also performed this test on 'intraspecific' clusters of individuals that might represent distinct taxonomical unities, and additional species from which the barcodes were available in our database, or in public databases (Genbank, BOLD, see Table 3[42]). Additionally, as an example on Little Penguins, we used the non-equilibrium coalescent approach implemented in the program IM, where an ancestral population splits into two constant-sized populations in the past and potentially exchange migrants [43]. Modal values of the population mutation parameter (θ), time of population divergence (tpop), time to the most recent common ancestor (TMRCA) and scaled migration rate (M) were obtained from the posterior distributions of these parameters using a Monte Carlo Markov Chain run for 12.26 million generations after a burnin of 100,000 generations.

### Assignment test

The correct assignment of individuals to species was performed in a decision-theoretic framework based on coalescent theory in Assigner [24]. The species selected had a ratio of among-species:maximum within-species genetic distances <10, and with N ≤ four individuals (Common Goldeneye, Lincoln's Sparrow, Sandwich Tern, and Gentoo Penguin). The *COI *sequence of one randomly selected individual was excluded from the matrix and used as the query sequence. For each of the sister species of the pair (target groups), the evolutionary parameter θ (twice the product of the female effective population size and neutral mutation rate) with corresponding maximum likelihood was estimated from the data in FLUCTUATE [44]. These values were used to calculate the likelihood of each of the target groups after re-including the query sequence to be assigned in Assigner [24].

### Distance and threshold estimation

Distances under the K2P model were calculated among sister-species and within-species in MEGA 3.1 [45]. Complete deletion was used in each comparison, to keep the number of base pairs equal in intra- and interspecific comparisons. Because the precision of the mtDNA barcode relies on the expectation that within-species variation is lower than among-species variation [1], the mean estimate of among species distances and the maximum value of pairwise intraspecific distances were used in the comparisons. The average level of intraspecific variation estimated across 260 species of birds of North America (0.27% of sequence divergence, yielding a threshold of 2.7% sequence divergence) [11] was used to test the efficacy of the 10 × rule in the sister-species pairs. To evaluate how variation in rates of evolution of *COI *in different lineages of birds [26] affect distance comparisons at sister-species levels, we selected six clades of birds for which divergence times have been estimated previously with relaxed clock methods (terns [46], shanks [47], alcids [48], penguins [49], and kiwis [50]). K2P distances of species pairs were plotted against divergence times, and *COI *distances between sister species of Terns, Shanks and Penguins were mapped on the corresponding chronograms.

## Authors' contributions

AJB and EST designed the scope of the research. EST carried out the lab work, data assembly, and analysis except for coalescent simulations in IM which were done by AJB. Both authors wrote and approved the final manuscript.

## Supplementary Material

Additional file 1 **Sister-species differences in *COI *barcode sequences**. Sister-species pairs and sampling, fixed substitutions (fixed subst.), bootstrap support, mean interspecific K2P distances (D_inter_), and maximum intraspecific K2P distance within each species (D_intra_). Click here for file

Additional file 2 **Neighbor-joining tree topology constructed from DNA barcodes of sister species of birds**. Neighbor-joining tree topology of ~650 bp of mitochondrial gene *COI*, under the K2P model and pairwise deletion. File in nexus format, opens in TreeView. Click here for file
